# Continuing medical education in China: evidence from primary health workers’ preferences for continuing traditional Chinese medicine education

**DOI:** 10.1186/s12913-023-10153-y

**Published:** 2023-11-03

**Authors:** Hao Yan, Zhaoran Han, Hanlin Nie, Wanjin Yang, Stephen Nicholas, Elizabeth Maitland, Weihan Zhao, Yong Yang, Xuefeng Shi

**Affiliations:** 1 School of Management, Beijing University of Chinese Medicine, Beijing, China; 2 Australian National Institute of Management and Commerce, Sydney, NSW Australia; 3 Guangdong Institute for International Strategies, Guangdong University of Foreign Studies, Guangzhou, China; 4 School of Economics and School of Management, Tianjin Normal University, Tianjin, China; 5 Newcastle Business School, University of Newcastle, Callaghan, NSW Australia; 6 University of Liverpool Management School, University of Liverpool, Liverpool, UK; 7 Medical Device Regulatory Research and Evaluation Centre, West China Hospital, Sichuan University, Chengdu, China; 8 National Institute of Traditional Chinese Medicine Strategy and Development, Beijing University of Chinese Medicine, Beijing, China

**Keywords:** Continuing medical education, Discrete choice experiment, Health workers, Learning preferences, Traditional Chinese Medicine technologies

## Abstract

**Background:**

Continuing Medical Education (CME) is an important part of the training process for health workers worldwide. In China, training in Traditional Chinese Medicine (TCM) not only improves the expertise of medical workers, but also supports the Chinese Government’s policy of promoting TCM as an equal treatment to western medicine. CME, including learning Traditional Chinese Medicine Technologies (TCMTs), perform poorly and research into the motivation of health workers to engage in CME is urgently required. Using a discrete choice experiment, this study assessed the CME learning preferences of primary health workers, using TCMT as a case study of CME programs.

**Methods:**

We conducted a discrete choice experiment among health workers in Shandong Province, Guizhou Province, and Henan provinces from July 1, 2021 to October 1, 2022 on the TCMT learning preferences of primary health workers. The mixed logit model and latent class analysis model were used to analyze primary health workers’ TCMT learning preferences.

**Results:**

A total of 1,063 respondents participated in this study, of which 1,001 (94.2%) passed the consistency test and formed the final sample. Our key finding was that there were three distinct classes of TCMT learners. Overall, the relative importance of the seven attributes impacting the learning of TCMTs were: learning expenses, expected TCMT efficacy, TCMT learning difficulty, TCMT mode of learning, TCMT type, time required to learn, and expected frequency of TCMT use. However, these attributes differed significantly across the three distinct classes of TCMT learners. Infrequent users (class 1) were concerned with learning expenses and learning difficulty; workaholics (class 2) focused on the mode of learning; and pragmatists (class 3) paid more attention to the expected TCMT efficacy and the expected frequency of TCMT use. We recommend targeted strategies to motivate TCMT learning suited to the requirements of each class of TCMT learners.

**Conclusion:**

Rather than a single TCMT medical education program for primary health workers, CME programs should be targeted at different classes of TCMT learners.

**Supplementary Information:**

The online version contains supplementary material available at 10.1186/s12913-023-10153-y.

## Background

Due to the complex and changing nature of medical knowledge, health workers undergo long periods of training, including university education and post-work practice. Previous studies have shown that four or more years of medical school education, and then several years of residency training, are both required to become a qualified physician in many countries, such as Germany, the United States, and China [1–3]. The advancement of medical practices, the updating of medical knowledge, and constant policy changes in the healthcare system, all of which mean health workers face tremendous learning challenges. Continuous learning and lifelong education ensure qualified health workers provide constantly improving medical services for patients [4]. Continuing medical education (CME) is any activity to maintain, develop, or increase their knowledge, skills, and professional performance and relationships that a physician uses to provide services for patients [5]. Besides keeping healthcare professionals’ knowledge and skills current and promoting more efficient use of health resources, physicians who do not participate in CME have lower persuasiveness in making clinical decisions [6–8]. CME has been shown to enhance the knowledge, skills, attitudes, and clinical outcomes of health workers [9, 10].

In China, CME is a mandatory requirement for continuous registration, requiring health workers to participate in CME activities each year and earn no less than 25 credits [11]. The content of CME includes new developments and advances in medicine, professional theories, treatment and management of common diseases, health policies and laws, new skills and practices and research capabilities. While CME has a long tradition in China, studies have shown that CME faces problems of outdated curriculum, lack of initiative in learning, only concerned with getting credits, short-term training resulting in unsystematic learning, and insufficient funding for grassroots CME [12–14].

In China, Traditional Chinese Medicine Technology (TCMT), which includes simple, convenient, cheap, and effective herbal prescriptions, acupuncture, massage, cupping and moxibustion [15], has broad-based patient support and is given equal emphasis with Western medicine in the Chinese government’s Healthy China 2030 Program and health policies [16]. Forming part of China’s CME, the promotion and use of TCMTs promotes the rational use of medical resources, improves the service capacity of TCM, and reduces the medical cost burden of residents [17, 18].

TCMT is an essential component of CME. It plays an important role in enhancing the capacity of TCM health services. The government has been very supportive of the TCMT and has been steadily increasing its support in recent years. The 2022 TCM Development Plan calls for clinical, dental and public health to complete the required TCMT CME, and for 100% of community health units and more than 80% of village health units to be able to provide TCM services [19]. However, we noticed that the CMT CME effect is not ideal in primary health institutions in some areas. For instance, Health workers lack knowledge about TCMT. A study conducted by Wang et al. found that only 41.8% of health workers in Shanghai’s community health units know TCMT [20]. Meanwhile, primary health workers have little interest in learning TCMT and are willing to spend time learning TCMT. A study by Shi et al. showed that 37.1% of health workers in the village were unwilling to learn TCMT [21]. Moreover, Li et al. also conducted a study which indicated that there was a low number of health workers who used TCMT in clinical practice within township hospitals [22]. So, we believe that the TCMT CME should fully consider the learning preferences of health workers.

Previous studies have mostly examined TCMT’s current status, requirements and influencing factors, with few studies examining the motivation to learn TCMTs from the perspective of health workers. This paper addresses how to improve primary health workers’ motivation to learn TCMTs through CME. Specifically, we use a discrete choice experiment (DCE) to investigate Chinese primary health workers’ preferences for learning TCMTs and to explore the incentive mechanisms for promoting TCMT learning.

## Methods

Developed by health economics, discrete choice experiments (DCEs) are one stated preference method used in applied economics to address key policy issues [23]. DCEs use a survey to quantitatively measure explicit preferences to determine a person’s or group’s trade-offs and choices for products and services under different hypothetical scenarios [23, 24]. Respondents choose between different scenarios that contain hypothetical alternatives, which are composed of different attributes and different levels of each attribute [25–27]. The different learning scenarios are designed, from which health workers then choose, to measure their preferences for learning TCMTs.

### Respondents

Purposive sampling and convenience sampling were used to gather the research sample. Based on the level of economic development and geographical characteristics of China, three provinces were selected for this study: Shandong Province (eastern region, high economic level), Henan Province (central region, medium economic level) and Guizhou Province (western region, low economic level). Then, 1–2 counties in each province were selected that were promoting the learning and use of TCMTs, i.e., Laizhou County in Shandong Province, Xiangfu County and Yuanyang County in Henan Province and Qingzhen County in Guizhou Province. Within the selected counties, county hospitals and township hospitals were selected for the study and each province ensured that the total number of hospitals selected was approximately the same. In Shandong, Henan and Guizhou provinces, 16, 17 and 12 hospitals respectively were selected for the study. We conducted the research in the selected primary healthcare units from July 1, 2021 to October 1, 2022. Using a combination of online and offline methods, the survey was completed anonymously. During the research, a face-to-face questionnaire survey was conducted with approximately 20–50% of the selected health workers on duty, totaling 1,063. The targeted respondents were healthcare workers who might use TCMTs in their treatment processes, including Conventional Medicine (CM) physicians, TCM physicians, and nurses.

### Survey tools

In our DCE study, the constituent properties of hypothetical scenarios and their levels were determined through a literature review and expert advice. First, a literature review was conducted to sort out the attributes that influence health workers’ motivation to learn TCMTs. We then invited 7 experts from the field of health research and management to form an advisory group to assess the attributes initially identified and their levels. After expert adjustment, Table 1 displays the seven attributes (learning expenses, learning difficulty, mode of learning, type of technology, expected technology efficacy, expected frequency of use, and time required to learn) [21, 28, 29] and their levels. Four attributes (learning expenses, learning difficulty, mode of learning, and time required to learn) focused on the sacrifices and efforts that healthcare workers were willing to make to learn the TCMTs, related to economy, time, and effort. The other three attributes (type of technology, expected technology efficacy, and expected frequency of use) represent the characteristics exhibited by TCMTs in clinical practice. The expected technology efficacy refers to the therapeutic effect in TCMT clinical practice compared with existing technologies; the type of technology refers to internal treatments that require internal medication, such as herbal prescriptions, or external treatment that do not require medication, such as acupuncture, massage, moxibustion and cupping; and the expected frequency of use refers to the frequency of application of learning TCMTs in diagnosis and treatment.

**Table 1:** Attributes, definitions and levels for DCE choice questions

Attributes	Definition	Levels
Learning expenses	Expenses paid by individuals for learning TCMTs.	0 yuan
		500 yuan
		800 yuan
		1200 yuan
Learning difficulty	The difficulty to master and use a TCMT for a person who does not know it.	Simple
		Normal
		Difficulty
Mode of learning	Whether to suspend work when learning TCMTs.	Off-the-job learning
		On-the-job learning
Type of technology	The therapeutic method of learning TCMTs.	Internal treatment
		External treatment
Expected technology efficacy	The therapeutic effect of learning TCMTs compared with existing techniques.	Higher than before
		Similar to before
		Uncertain efficacy
Expected frequency of use	Frequency of application of learning TCMTs in diagnosis and treatment.	40%
		30%
		20%
Time required to learn	Learning time required to master a TCMT.	7 days
		14 days
		21 days

Given the above attributes and levels, the respondents’ burden of answering a full factorial design comprised 1,296 ($={2}^{2}\times {3}^{4}\times 4$) hypothetical scenarios, which in turn would lead to 839,160 ($=(\text{1,296}\times \text{1,295})/2$) group selection tasks. The D-efficient design in SAS 9.2 addressed this problem by using an orthogonal design to generate 18 representative choice sets, which were randomly assigned to three versions of the questionnaire. Respondents were asked to choose which of the two hypothetical techniques they would prefer to learn, with Table 2 providing a representative example of the selection tasks. Additional data on age, sex, occupation, job title, and respondents’ self-assessed scores of motivation to learn medical technologies were collected.

**Table 2:** An example of a DCE choice set

Attributes	Technology A	Technology B
Learning expenses	0 RMB	800 RMB
Learning difficulty	Simple	Normal
Mode of learning	Off-the-job learning	On-the-job learning
Type of technology	External treatment	Internal treatment
Expected technology efficacy	Uncertain efficacy	Higher than before
Expected frequency of use	40%	30%
Time required to learn	7 days	14 days
Which technology do you prefer?	▢	▢

### Quality control

In order to better control the quality of responses, a consistency check question with a clearly superior option was added in every questionnaire to assess whether respondents took the survey seriously. Responses that failed the consistency test were precluded in our research. Before the deployment of the study, we conducted a pilot survey and adjusted the wording and layout to make the questionnaire clearer and easier to understand. During the on-site survey, trained investigators were assigned to provide questionnaire instructions and assistance in answering any questions from respondents during the survey.

### Theory and statistical analyses

The theoretical basis of DCE random utility theory [30], which states that when a decision-maker facing with a choice, his/her preferences for a particular choice can be described by the utility value of the chosen object. Using a mixed logit model, the learning preferences of health workers, or the utility of learning a TCMT, was specified:

1 $$\begin{array}{l}{U_{ijt}} = {\beta _{1n}}Expense{s_{njt}} + {\beta _{2n}}Difficult{y_{njt}} +\\\,\,\,\,{\beta _{3n}}Mod{e_{njt}} + {\beta _{4n}}Typ{e_{njt}} + {\beta _{5n}}Efficac{y_{njt}} +\\\,\,\,\, {\beta _{6n}}Frequenc{y_{njt}} + {\beta _{7n}}Tim{e_{njt}} + {\varepsilon _{njt,}}\end{array}$$

where U is the utility (U) participant i acquires from choosing TCMTs, j for choice set t, ɛ is participant-specific random error and incorporates both preferences estimates and variance-scale for the respective treatment characteristics.

The latent class analysis (LCA) model assumes that participants have different preferences and that participants can be probabilistically grouped according to different preference classes, each corresponding to a unique pattern of learning preferences. Also, the LCA model assumes that the distribution of coefficients is discrete rather than continuous. Suppose that all participants are classified into Q classes and the utility of individuals n in Q classes choosing TCMTs j under choice set t is

2 $${U}_{njt}={\beta }_{q}^{{\prime }}{X}_{njt} + {\epsilon }_{njt},$$

where ${\beta }_{q}$ is a class-specific parameter vector, and the other variables have the same meaning as in Eq. (1).

Akaike information criterion (AIC) and Bayesian information criterion (BIC) are used to select the best-fit model from a finite set of models for a given set of data, with smaller values indicating a better model fit [31]. The AIC derives from information theory and aims to select the model that generates the probability distribution with the lowest deviation from the actual one. The BIC is calculated through an asymptotic approximation from a large sample to perform a full comparison of Bayesian models [32]. We calculated AIC and BIC values that showed that the three classes model was optimal.

Relative importance is the ratio of the range of utilities within an attribute to the sum of the ranges of utilities of all attributes. The relative importance of each attribute is calculated by finding the maximum utility difference between attribute levels and is expressed as a percentage of the sum of all maximum differences [33]: $\frac{{{\beta }}_{q} max}{{{\sum }_{n}^{m}{\beta }}_{m max}}$, where ${{\beta }}_{q max}$refers to the maximum value of the coefficient of attribute q and the denominator is the sum of the maximum values of the coefficients of all attributes.

Uptake rate prediction analysis, a very flexible post-evaluation tool, is a simple way to describe how the uptake probability changes with the change of attribute and also provides a way to simulate interesting scenarios. The logit probability estimation equation of the individual choosing one scenario instead of another is:

3 $${\text{P}}_{{j}_{1}} = \frac{\text{e}{\beta }1\times 1{\text{j}}_{1}+{\beta }2\times 2{\text{j}}_{1}+...+{\beta }\text{n}\times \text{n}{\text{j}}_{1}}{\sum \text{e}{\beta }1\times 1{\text{j}}_{2}+{\beta }2\times 2{\text{j}}_{2}+...+{\beta }\text{n}\times \text{n}{\text{j}}_{2}} \forall {\text{j}}_{1}, {\text{j}}_{2}\in \text{J,}$$

where ${Xnj}_{1}$ and ${Xnj}_{2}$ were the attribute coefficient vectors of alternative ${j}_{1}$ and alternative ${j}_{2}$, respectively.

All analyses were performed in Stata 16.0, using the default (uninformative) priors for the MIXL model and the lclogit2 procedure for the latent class models.

## Results

We conducted a face-to-face anonymous survey in Shandong, Henan and Guizhou provinces. A total of 1,063 questionnaires were collected from TCM physicians, CM physicians, and nurses, of which 1,001 (94.2%) were valid, which comprised the study sample.

The mean age of the respondents was 36.6 years (SD = 9.4), and the median of years in the profession was 10 years (IQR: 4 years—20 years); 34.9% of the respondents were male; 32.4% of respondents were CM physicians, 31.5% were TCM physicians and 36.2% were nurses. Half of the respondents had a junior job title (50.9%) and more than half had a bachelor’s degree or higher (58.9%).

### Aggregate results

Table 3 displays the result of the mixed logit model, where all the attributes had a significant effect on the learning preferences. Respondents showed a preference for a higher expected efficacy (β: 1.088, OR: 2.969) and a similar expected efficacy (β: 0.313, OR: 1.367) over an uncertain expected efficacy technology. Preferences for simple learning difficulty (β: 0.693, OR: 2.000) and normal difficulty (β: 0.513, OR: 1.670) dominated a difficult TCMT. Respondents also preferred on-the-job learning to off-the-job learning (β: 0.575, OR: 1.776) and external treatments were preferred by respondents over internal treatments (β: 0.432, OR: 1.540). In addition, Table 3 reports that learning expenses (β: -0.0013, OR: 0.999), expected frequency to use the technology (β: 0.0114, OR: 1.011) and time required to learn (β: -0.0217, OR: 0.979) were all significant attributes that influenced respondents’ preferences to learn TCMTs. Overall, respondents preferred an external treatment technology that is less costly, less time-consuming, less difficult to learn, can be delivered through on-the-job learning and can be applied in daily work.

**Table 3:** Mixed logit model results

Attributes	β	OR	95% CI		SE	SD	SE
Learning expenses (by yuan)	-0.0013***	0.999	0.998	0.999	0.0001	0.0014***	0.0001
Learning difficulty (ref: Difficult)							
Simple	0.693***	2.000	1.759	2.274	0.066	0.332	0.184
Normal	0.513***	1.670	1.472	1.894	0.064	0.054	0.240
Mode of learning (ref: Off-the-job learning)							
On-the-job learning	0.575***	1.776	1.584	1.992	0.058	1.012***	0.090
Type of technology (ref: Internal treatment)							
External treatment	0.432***	1.540	1.384	1.715	0.055	0.806***	0.089
Expected technology efficacy (ref: Uncertain efficacy)							
Higher than before	1.088***	2.969	2.516	3.504	0.085	0.927***	0.107
Similar to before	0.313***	1.367	1.208	1.547	0.063	0.008	0.194
Expected frequency of use (by 1%)	0.0114**	1.011	1.005	1.018	0.0032	0.0382***	0.0069
Time required to learn (by day)	-0.0217***	0.979	0.971	0.986	0.0040	0.0011	0.0163
AIC	6990.42						
BIC	7123.51						
Log likelihood	-3477.21						
Respondents, n	1,001						
Observations, n	12,012						

β = coefficient; OR = Odds ratio; 95% CI: 95% confidence interval SE = standard error; SD = standard deviation;

ref = reference; AIC = Akaike information criterion; BIC = Bayesian information criterion;

*P < 0.05, **P < 0.01, ***P < 0.001

### Results by class

#### Model estimate

**Fig. 1:**
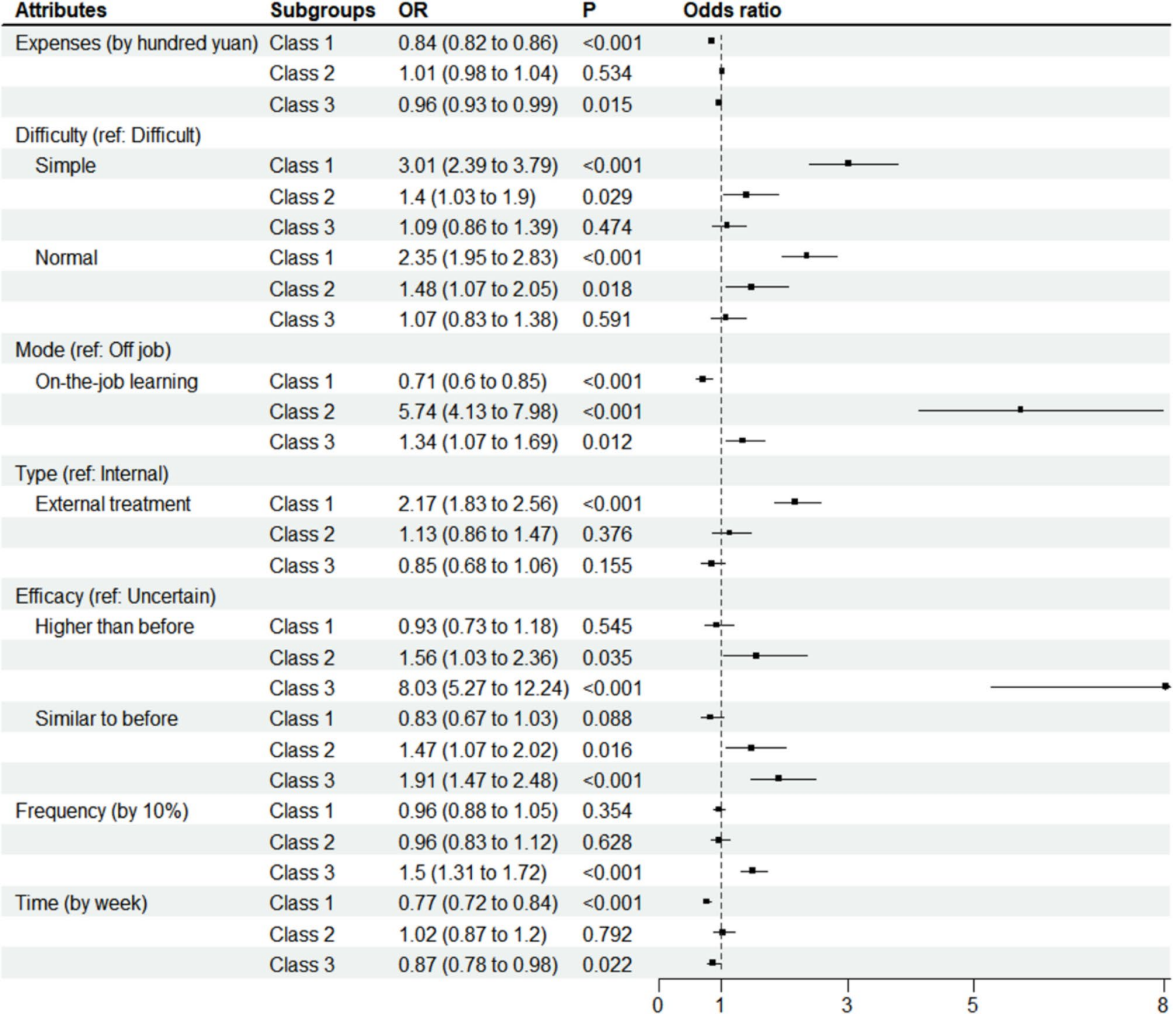
Latent class results of discrete choice experiments

According to BIC in Table 4, the latent class analysis identified three classes of respondents with different preferences. We assigned the class with the highest probability as the latent class. Table 5 reports that there were statistically significant differences between the three classes in terms of years in the profession, sex, occupation, and self-assessed learning motivation score. As displayed in Table 5; Fig. 1, class 1 accounted for the biggest proportion of respondents (50.1%), showing a strong preference for TCMTs with simple learning difficulty (OR: 3.01) and external treatment (OR: 2.17), with respondents in class 1 having significantly lower learning motivation scores and the lowest percentage of TCM physicians. Class 2 accounted for the smallest proportion of respondents (20.1%), showing a strong preference for on-the-job learning (OR: 5.74), with respondents having the longest years in the profession. Class 3 consisted of 29.8% of respondents and showed a strong preference for higher efficacy (OR: 8.03) and higher usage frequency (OR: 1.5), with the highest proportion of TCM physicians.

**Table 4:** Criteria for model selection

Number of Classes	BIC	AIC
1	7123.505	6990.419
2	7149.267	7002.005
3	7014.768	6774.239
4	7019.883	6686.088
5	7086.932	6659.870

Akaike Information Criterion (AIC) and Bayesian Information Criterion (BIC)

**Table 5:** Demographic characteristics for respondents by class (n = 1001)

	Class 1	Class 2	Class 3	P value
	502 (50.1%)	201 (20.1%)	298 (29.8%)	
Age, median (IQR)	35 (29–43)	37 (31–44)	34 (29–43)	0.060
Years in the profession, median (IQR)	10 (4–19)	10 (6–20)	9 (3–19)	0.008
Male (N, Percent)	161 (32.1%)	62 (30.8%)	126 (42.3%)	0.006
Occupation				
CM physicians	182 (36.3%)	64 (31.8%)	78 (26.2%)	< 0.001
TCM physicians	97 (19.3%)	50 (24.9%)	168 (56.4%)	
Nurses	223 (44.4%)	87 (43.3%)	52 (17.4%)	
Education				
≥Undergraduate	282 (56.2%)	117 (58.2%)	191 (64.1%)	0.086
≤ Junior college	220 (43.8%)	84 (41.8%)	107 (35.9%)	
Job title				
Senior title	37 (7.4%)	21 (10.4%)	34 (11.4%)	0.269
Intermediate title	169 (33.6%)	76 (37.8%)	96 (32.2%)	
Junior title	268 (53.4%)	90 (44.8%)	152 (51.0%)	
Other job title	28 (5.6%)	14 (7.0%)	16 (5.4%)	
Learning motivation score, median (IQR)	8 (6–10)	10 (7–10)	9 (7–10)	0.003

Each of the classes had its preferred characteristics and demographic characteristics, which means that each of different classes requires different policies to motivate TCMTs learning.

#### Relative importance

**Fig. 2:**
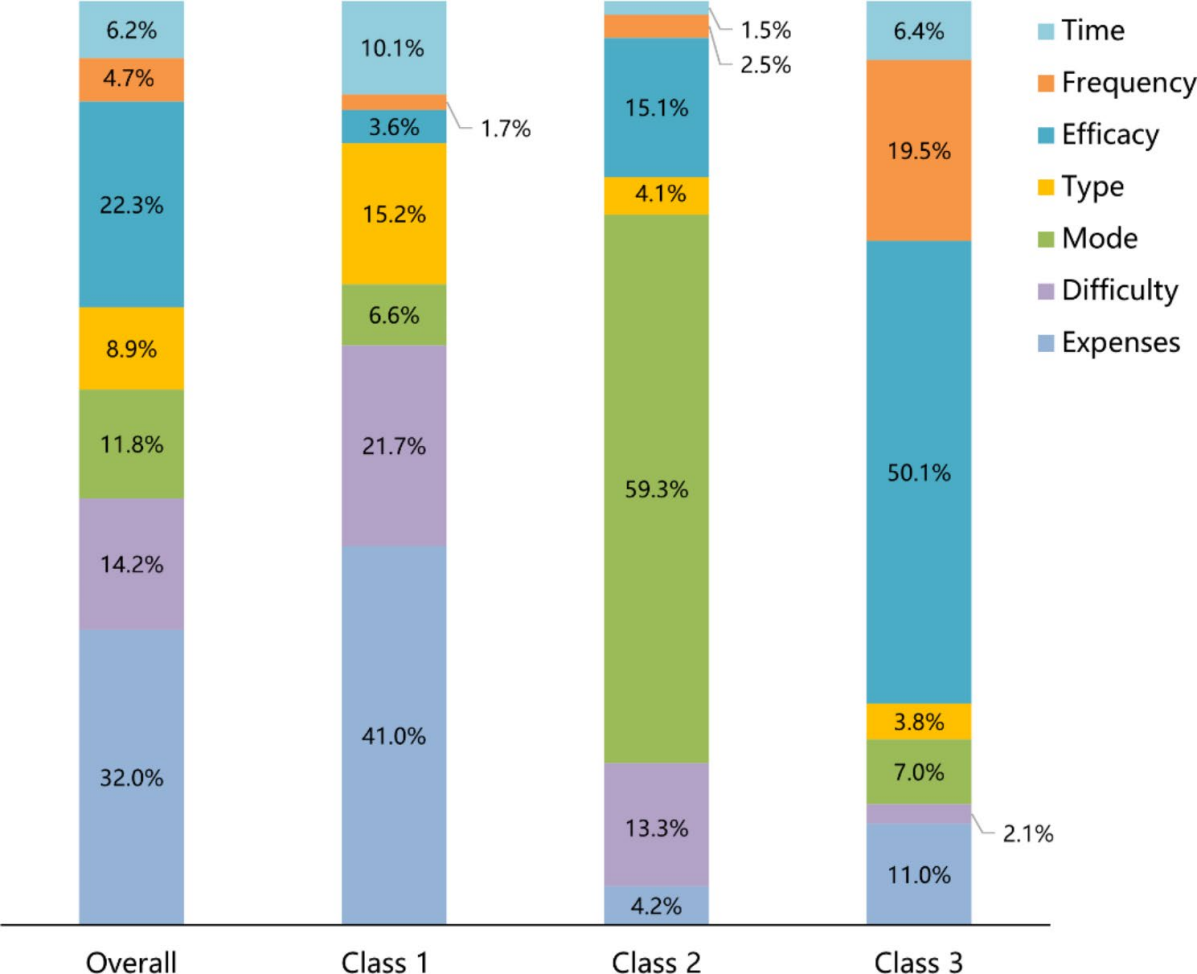
Relative importance by class

Overall, the attribute with the highest relative importance was learning expenses (32.0%), followed by expected efficacy of TCMTs (22.3%), learning difficulty (14.2%), mode of learning (11.8%), type of TCMTs (8.9%), the time required to learn (6.2%) and expected frequency of use (4.7%). As shown in Fig. 2, each class had a distinct pattern of the relative importance of attributes, which we classified as infrequent users (class 1) (n = 502, 50.1%), workaholics (class 2) (n = 201, 20.1%), and pragmatists (class 3) (n = 298, 29.8%). To be specific, among the infrequent users (class 1), learning expense had the highest relative importance (41.0%), followed by learning difficulty (21.7%), type of TCMTs (15.2%), the time required to learn (10.1%), mode of learning (6.6%), expected efficacy of TCMTs (3.6%) and expected frequency of use (1.7%). The relative importance of the mode of learning was the highest among workaholics (class 2) (59.3%), followed by expected efficacy of TCMTs (15.1%), learning difficulty (13.3%), learning expense (4.2%), type of TCMTs (4.1%), expected frequency of use (2.5%) and time required to learn (1.5%). Among the pragmatists (class 3), the expected efficacy of TCMTs had the highest relative importance (50.1%), followed by expected frequency of use (19.5%), learning expense (11.0%), mode of learning (7.0%), the time required to learn (6.4%), type of TCMTs (3.8%) and learning difficulty (2.1%).

#### Uptake rate

**Fig. 3:**
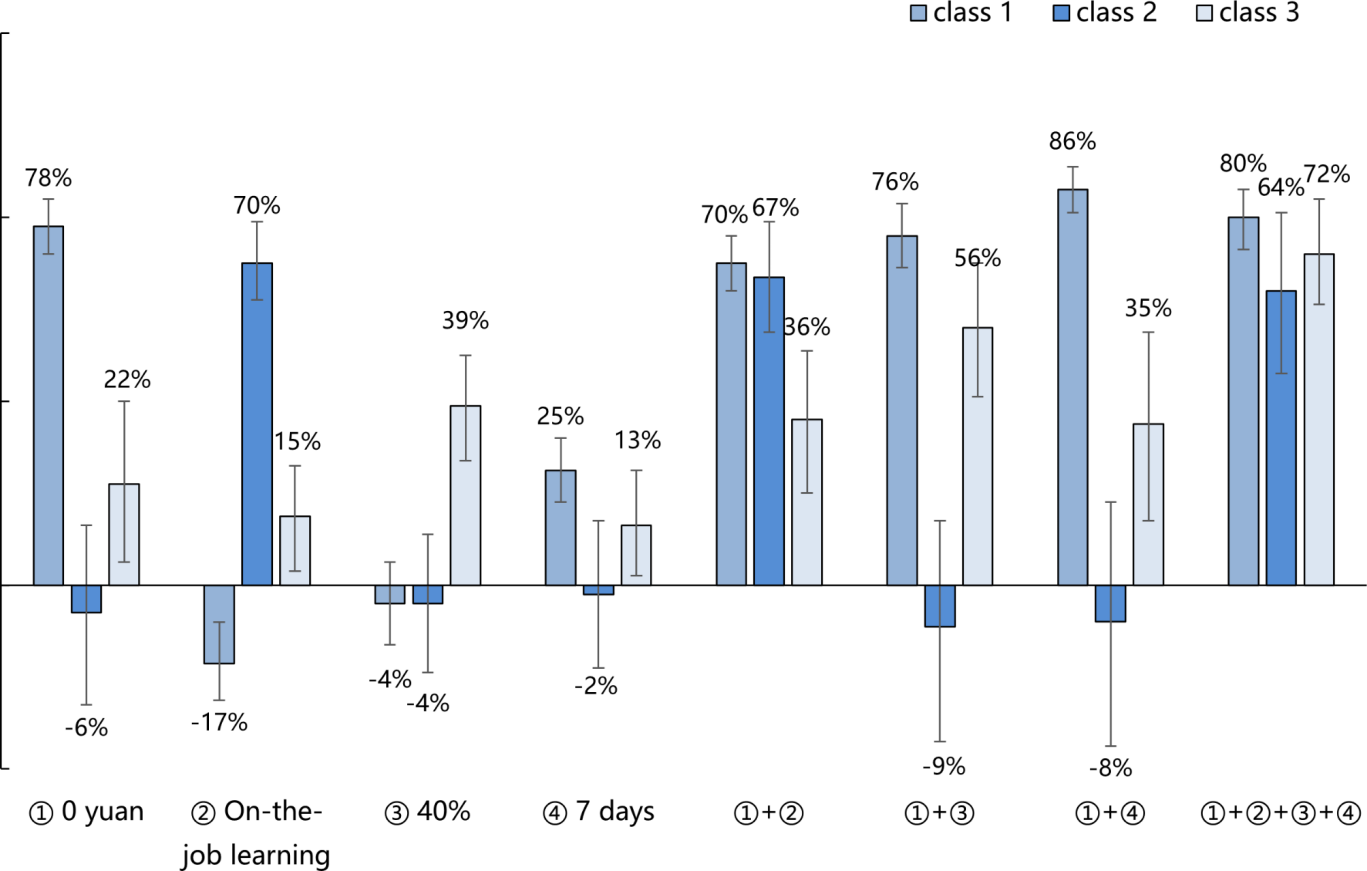
Uptake rate for TCMTs learning under various potential policy scenarios *Baseline learning TCMTs: learning expenses “1,200 yuan”; learning difficulty “normal”; mode of learning “off-the-job learning”; type of technology “internal treatment”; expected technology efficacy “similar to before”; expected frequency of use “20%”; time required to learn “7 days”

Based on the latent class analysis, we predicted the possible change in the uptake rate for TCMTs learning under different potential policy scenarios in Fig. 3. We only varied the level of attributes that can be changed by external factors: learning expenses, mode of learning, expected technology efficacy, and time required to learn. For infrequent users (class 1), the uptake rate was increased by 78% compared with the baseline when learning the TCMT was free; increased by 25% when shortening the time of learning to 7 days; and combining both, the uptake rate increased by 86%. For workaholics (class 2), the uptake rate was increased by 70% compared with the baseline when they can learn the TCMT without suspending their jobs. None of the other measures and their combinations lead to a higher uptake rate. For pragmatists (class 3), the uptake rate was increased by 39% compared with the baseline when the learned TCMT can be used 40% more frequently in daily health work; increased by 22% when learning the TCMT was free; increased by 15% when they could learn TCMT without leaving their jobs; and increased by 13% when the time required to learn was reduced to 7 days. When all the above scenarios were met simultaneously, the uptake rate increased by 72%.

## Discussion

Research on how to motivate health workers to maximize CME performance is rare. To assess the motivation for CME, we used learning TCMTs as an example. The Chinese government promotes TCM as an equal treatment to western medicine with official health policy fostering TCMTs in rural and urban areas as a safe, effective, convenient, and affordable primary healthcare services [34]. TCMT CME is fundamental to advancing the Chinese government’s commitment to TCM, but TCMTs learning in China has been unsatisfactory [15, 21]. Conducting an anonymous face-to-face survey of health workers in primary care institutions in three provinces, we found that taking all health workers together, health workers were motivated to learn TCMT when TCMT learning was less costly, less time-consuming, less difficult to learn, and could be learned on the job and used in their daily work. But, taking different classes of health workers, the motivations for TCMT differ across different types of learners.

Turning first to all health workers. the attribute of the highest relative importance was learning expenses (32.0%). In general, primary care workers consider their salaries to be relatively low [35, 36], which makes them hesitant to pay to learn a new technology with which they are not familiar. Learning expenses were followed by the expected efficacy of TCMTs (22.3%), learning difficulty (14.2%), mode of learning (11.8%), type of TCMTs (8.9%), time required to learn (6.2%) and expected frequency of use (4.7%). Consistent with the evidence that innovative learning technology is more likely to be adopted by university teachers when it is perceived as superior to existing tools, easy to use, and readily available [37], our results show that primary health workers were more to undertake CME when the expected efficacy and expected frequency of use of TCMTs was higher than already learned technologies with the same effects. Most Chinese primary care physicians have a low level of education relative to Chinese physicians generally [38], which meant that easy-to-learn technology was more acceptable for these primary health workers. For health workers who are very busy in their daily practice, then on-the-job training or a less time-consuming technology is more popular among these primary care workers. The possible explanation for health workers’ preferences for TCM external treatment technology over TCM internal treatment is that they were more proficient in conventional medicine and had less knowledge of TCM, since TCM internal treatment requires greater theoretical knowledge than TCM external treatments.

Our key finding comes from our latent class analysis model that revealed significant differences in learning motivations according to three main TCMT learner classes: infrequent users (50.1%), workaholics (20.1%) and pragmatists (29.8%). The three groups differed in their socio-demographic characteristics, and required different motivational learning strategies. We recommend CME trainers target different CME approaches to address each of these types of TCMT learners. Infrequent users had significantly lower learning motivation scores and the lowest percentage of TCM physicians. The attributes they valued most were learning expenses and the difficulty of the TCMTs to learn.

The infrequent users group had significantly lower learning motivation scores and the lowest percentage of TCM physicians. The attributes they valued most were learning expenses and the difficulty of the TCMTs learning. This class mostly consisted of CM physicians and nurses who used mainly conventional medical technologies in their daily work; had a weak basic theory of TCM [39]; sought TCMTs that were easy to learn; and preferred TCMT that had low learning expenses. There exists pressure on CM physicians to respond to the national policy to learn TCM technologies [40], however, improving CM physicians’ knowledge of TCM has a positive effect on their ability to provide integrative medical services [41]. For the infrequent user group, we can reduce the learning expenses of TCM learning, select simple TCMTs, and at the same time enrich the teaching mode to increase their interest in learning. By applying targeted learning approaches to the infrequent user group, the motivation of learning TCMTs and the willingness to apply TCMTs in clinical practice in this group can both be increased [42].

The workaholics, who accounted for 20.1% of all respondents, had the longest work years and the attribute they valued most was the mode of learning. Workaholics’ long stay of work implies that they have mastered the necessary medical skills to perform their daily procedures well, so they prefer learning CME on the job, which is consistent with research that found many health workers preferred on-the-job learning due to the conflict between work and learning [43]. This group values small group educational meetings, multidisciplinary discussions (both formal and informal) and interactive workshops, in particular, all of which have been shown to be effective measures of CME [44], and allow for on-the-job learning.

The third class was the pragmatists that contained the highest proportion of TCM physicians, who attached importance on the efficacy and expected frequency of TCMT use, and health workers of this class were more likely to be the actual users of TCMTs. They were extremely passionate about TCM, had a good theoretical foundation in TCM, and could better apply TCMTs learned through CME in practice, so pragmatists attach importance to TMCT frequency of use and efficacy, and expect that TCMTs should achieve more effective results for their patients [45]. For this group, it is necessary to screen learning programs to focus on the most effective TCMTs and to match TCMT learning to the urgent needs of the public [46, 47].

This study has several limitations. Our DCE study focused on seven attributes, but future studies should consider other attributes, such as TCMT equipment conditions and personal development opportunities, which also affect health workers’ TCMT learning preferences. DCEs measure stated preferences, which may differ from their actual behaviors. Further research is needed to expand the measures of primary health care workers’ revealed preferences. This study focused on the learning preferences of primary health workers for TCMTs, which can increase learners’ motivation and improve the quality of learning from a personal subjective perspective, but the data in this study did not analyze the actual objective learning effects, and further research on this topic is needed. Our recommendations apply to primary health workers, and future studies should explore CME for hospital health workers.

## Conclusion

TCMT CME is a key driver of the Chinese government’s commitment to promote TCM as an equal treatment with western medicine. Our study reveals the preferred CME approaches and recommends tailored TMCT CME programs. The key finding is that health workers can be divided into three distinct classes of TCMT learners. Infrequent users (class 1) (50.1%) preferred to learn simple and external treatment TCMTs; the workaholics (class 2) 20.1% preferred to learn TCMT on-the-job; and the pragmatists (class 3) 29.8% showed a strong preference for learning TCMTs with higher efficacy and more frequent use. We recommend that different measures can be taken to incentivize the three classes identified: for infrequent users (class 1), healthcare units can offer more free and short-learning in-hospital TCMT CME to increase their knowledge and interest in TCMT; for workaholics (class 2), it is crucial that TCMT CME does not disrupt their regular work; for pragmatists (class 3), healthcare units can offer more free, short-learning and frequently used TCMTs and teach them without disrupting normal work. While we used the example of learning TCMTs for our study, our research conclusions have implications for other CME programs.

### Electronic supplementary material

Below is the link to the electronic supplementary material.

Supplementary Material 1

## Data Availability

The data used and/or analyzed during the study are available from the corresponding author upon reasonable request.

## Abbreviations

CME: Continuing Medical Education
TCM: Traditional Chinese medicine
TCMTs: Traditional Chinese Medicine Technologies
DCEs: Discrete choice experiments
CM: Conventional Medicine
LCA: The latent class analysis
AIC: Akaike information criterion
BIC: Bayesian information criterion
