# Supplementary material for: Continuing medical education in China: evidence from primary health workers’ preferences for continuing traditional Chinese medicine education
Source: BMC Health Serv Res. 2023 Nov 3;23:1200. doi: 10.1186/s12913-023-10153-y (PMC10623727; doi:10.1186/s12913-023-10153-y)
Supplement: Supplementary file 1 — Supplementary Material 1 [file 12913_2023_10153_MOESM1_ESM.pdf]

Questionnaire on the Learning and Use of TCMTs among Rural and Primary Medical Workers (Version 1)

Dear Expert,

We are the research team of the National Natural Science Foundation of China project "Research on the Diffusion Incentive Mechanism of Appropriate Technologies for Traditional Chinese Medicine Based on Discrete Selection Experiments (Project No. 72074032)". This expert consultation is mainly from the perspective of medical workers to understand your problems and needs in learning and using traditional Chinese medicine technologies (TCMTs), so as to put forward constructive opinions and suggestions for the effective promotion of TC. In this study, "appropriate health technologies" refers to a series of safe, effective, economical, and practical health and TCM appropriate technologies that are suitable for basic medical use, can be learned and used. Among them, "appropriate TCM technologies" refers to the diagnosis, treatment technologies, medical prescriptions and drug combinations that are suitable for common and frequently occurring diseases at the grass-roots level, easy to operate, and easy to be mastered and used by primary medical workers, such as acupuncture, massage, cupping, and Chinese herbal formulas and so on.

We solemnly promise that this survey is for academic research only and will not be used for other purposes. Meanwhile, the questionnaire does not involve any personal privacy, so please rest assured to fill it out. We sincerely thank you for your strong cooperation!

Part 1: Basic information

1.1 Your gender is ______.

①male ②female

1.2 Your age is ______.

1.3 Your education level is ______.

①postgraduate ②undergraduate ③junior college ④technical secondary school and technical school ⑤high school ⑥junior high school and below

1.4 Your professional title is ______.

①senior title ②associate senior title ③intermediate title ④junior title ⑤other job title ______

1.5 Your type of practice is ______.

① conventional medicine practicing (assistant) physician ② traditional Chinese medicine practicing (assistant) physician ③ public health practicing (assistant) physician ④ practicing (assistant) nurse ⑤other______

1.6 Your monthly income is ______ yuan.

1.7 Your expected monthly income is ______ yuan.

1.8 The type of your institution is ______.

①county hospital ②county hospital of traditional Chinese medicine ③township hospital ④

community health service center ⑤community health service station ⑥village clinic ⑦others__

1.9 Your department is ______.

① internal medicine department ② surgery department ③ traditional Chinese medicine department ④gynecology department ⑤pediatric department ⑥other department ______

1.10 You have been in this profession for ______ year (years).

1.11 Your establishment status is ______.

①within the establishment ②contract system ③temporary employment ④re-employment

1.12 You have ______ hour (hours) of free time outside of work every day, of which ______ hour (hours) are used for self-directed learning.

1.13 Your contact number is ________.

Part 2: Appropriate health technology learning and satisfaction

2.1 Have you participated in training on appropriate health technologies in the past 3 years? ________

①Yes (please continue to answer)

②No (please skip to question 2.8)

2.2 What appropriate health technologies training have you participated in in the past 3 years? (Multiple choices are available.) ________

① Traditional Chinese medicine technologies training ② Appropriate western medicine technologies training ③Appropriate public health technologies training ④Other (please specify) ________

2.3 The cumulative training time is ________ day (days), of which the training time for appropriate TCM technologies is ________ day (days).

2.4 In the past 3 years, you have learned a total of ______ appropriate technology (technologies), including ______ appropriate TCM technology (technologies).

2.5 The ways of training you receive include ________. (Multiple choices are available.)

①online training ②going out for further study ③going out for centralized classes ④experts coming to the hospital for guidance ⑤other ________

2.6 How is the overall effect of your learning technology? ________

①Very good ②Relatively good ③Average ④Relatively poor ⑤Very poor

2.7 How satisfied are you with the promotion of appropriate health technologies in primary medical institutions? (Satisfaction level is represented by 11 numbers ranging from 0 to 10. 0 means very dissatisfied, and 10 means very satisfied. Please tick √ on the scale describing your satisfaction with the promotion.)

0 1 2 3 4 5 6 7 8 9 10

（very dissatisfied） （very satisfied）

2.8 Whether the institution has a reward mechanism for appropriate technologies learning?

①Yes (please continue to answer) ②No (please skip to question 2.10)

2.9 What kind of reward system for appropriate technologies learning does your institution have? (Multiple choices are available.) ________

①Bonus ②Professional title promotion ③Continuing education credits ④Other (please specify) ________

2.10 How much do you know about appropriate health technologies? ________

①know very well ②know more ③generally know ④do not know ⑤do not know at all

2.11 How motivated are you to learn appropriate health technologies? (The level of learning motivation is represented by 11 numbers ranging from 0 to 10. 0 indicates no willingness to learn, and 10 indicates a great willingness to learn. Please tick √ on the scale that describes your enthusiasm for technology learning.)

0 1 2 3 4 5 6 7 8 9 10

（very reluctant） （very willing）

Part 3: Investigation of preferences for learning TCM technologies

Suppose that you have two TCM technologies to learn now, technology A and technology B. The two technologies differ in terms of learning expenses, learning difficulty, mode of learning, type of technology, expected technology efficacy, expected frequency of use and time required to learn. In the following 7 pairs of choices, please choose the TCM technology that you prefer based on your personal preferences. When making your choice, please assume that except for the seven attributes listed, the other conditions for technology A and technology B are the same.

3.1 If you choose one of the following pairs of TCM technologies (option A and option B), which one would you prefer to learn? (Tick √ in the box to the right of your selected technology.)

attribute of technology	learning expenses	learning difficulty	mode of learning	type of technology	expected technology efficacy	expected frequency of use	time required to learn	Which technology do you prefer?
technology A	0 yuan	difficult	on-the-job learning	internal treatment	uncertain efficacy	40%	14 days	
technology B	1200 yuan	simple	off-the-job learning	external treatment	similar to before	30%	21 days	

If there is a TCM technology that you tend to choose in real life, will you choose to learn it?

3.2 If you choose one of the following pairs of TCM technologies (option A and option B), which one would you prefer to learn? (Tick √ in the box to the right of your selected technology.)

attribute of technology	learning expenses	learning difficulty	mode of learning	type of technology	expected technology efficacy	expected frequency of use	time required to learn	Which technology do you prefer?
technology A	500 yuan	simple	on-the-job learning	external treatment	higher than before	40%	7 days	
technology B	0 yuan	normal	off-the-job learning	internal treatment	similar to before	20%	14 days	

If there is a TCM technology that you tend to choose in real life, will you choose to learn it?

3.3 If you choose one of the following pairs of TCM technologies (option A and option B), which one would you prefer to learn? (Tick √ in the box to the right of your selected technology.)

attribute of technology	learning expenses	learning difficulty	mode of learning	type of technology	expected technolog y efficacy	expected frequency of use	time required to learn	Which technology do you prefer?
technology A	500 yuan	difficult	off-the-job learning	external treatment	similar to before	20%	21 days	
technology B	800 yuan	simple	on-the-job learning	internal treatment	uncertain efficacy	30%	7 days	

If there is a TCM technology that you tend to choose in real life, will you choose to learn it?

3.4 If you choose one of the following pairs of TCM technologies (option A and option B), which one would you prefer to learn? (Tick √ in the box to the right of your selected technology.)

attribute of technology	learning expenses	learning difficulty	mode of learning	type of technology	expected technology efficacy	expected frequency of use	time required to learn	Which technology do you prefer?
technology A	0 yuan	simple	off-the-job learning	internal treatment	higher than before	20%	14 days	
technology B	500 yuan	normal	on-the-job learning	external treatment	uncertain efficacy	40%	21 days	

If there is a TCM technology that you tend to choose in real life, will you choose to learn it?

3.5 If you choose one of the following pairs of TCM technologies (option A and option B), which one would you prefer to learn? (Tick √ in the box to the right of your selected technology.)

attribute of technology	learning expenses	learning difficulty	mode of learning	type of technology	expected technology efficacy	expected frequency of use	time required to learn	Which technology do you prefer?
technology A	0 yuan	normal	off-the-job learning	external treatment	uncertain efficacy	30%	7 days	
technology B	800 yuan	difficult	on-the-job learning	internal treatment	higher than before	40%	14 days	

If there is a TCM technology that you tend to choose in real life, will you choose to learn it?

3.6 If you choose one of the following pairs of TCM technologies (option A and option B), which one would you prefer to learn? (Tick √ in the box to the right of your selected technology.)

attribute of technology	learning expenses	learning difficulty	mode of learning	type of technology	expected technology efficacy	expected frequency of use	time required to learn	Which technology do you prefer?
technology A	800 yuan	normal	on-the-job learning	external treatment	similar to before	40%	21 days	
technology B	500 yuan	difficult	off-the-job learning	internal treatment	higher than before	30%	7 days	

If there is a TCM technology that you tend to choose in real life, will you choose to learn it?

3.7 If you choose one of the following pairs of TCM technologies (option A and option B), which one would you prefer to learn? (Tick √ in the box to the right of your selected technology.)

attribute of technology	learning expenses	learning difficulty	mode of learning	type of technology	expected technology efficacy	expected frequency of use	time required to learn	Which technology do you prefer?
technology A	0 yuan	simple	off-the-job learning	internal treatment	higher than before	40%	7 days	
technology B	1200 yuan	difficult	off-the-job learning	internal treatment	uncertain efficacy	20%	28 days	

If there is a TCM technology that you tend to choose in real life, will you choose to learn it?
